# Characterization of Cereulide Synthetase, a Toxin-Producing Macromolecular Machine

**DOI:** 10.1371/journal.pone.0128569

**Published:** 2015-06-04

**Authors:** Diego A. Alonzo, Nathan A. Magarvey, T. Martin Schmeing

**Affiliations:** 1 Department of Biochemistry, McGill University, Montréal, QC H3G 0B1, Canada; 2 Department of Chemistry & Chemical Biology, McMaster University, M.G. DeGroote Institute for Infectious Disease Research, 1200 Main St. W, Hamilton, Ontario L8N 3Z5, Canada; 3 Groupe de Recherche Axé sur la Structure des Protéines (GRASP), McGill University, Montréal, QC H3G 0B1, Canada; University Paris South, FRANCE

## Abstract

Cereulide synthetase is a two-protein nonribosomal peptide synthetase system that produces a potent emetic toxin in virulent strains of *Bacillus cereus*. The toxin cereulide is a depsipeptide, as it consists of alternating aminoacyl and hydroxyacyl residues. The hydroxyacyl residues are derived from keto acid substrates, which cereulide synthetase selects and stereospecifically reduces with imbedded ketoreductase domains before incorporating them into the growing depsipeptide chain. We present an *in vitro* biochemical characterization of cereulide synthetase. We investigate the kinetics and side chain specificity of α-keto acid selection, evaluate the requirement of an MbtH-like protein for adenylation domain activity, assay the effectiveness of vinylsulfonamide inhibitors on ester-adding modules, perform NADPH turnover experiments and evaluate *in vitro* depsipeptide biosynthesis. This work also provides biochemical insight into depsipeptide-synthesizing nonribosomal peptide synthetases responsible for other bioactive molecules such as valinomycin, antimycin and kutzneride.

## Introduction

**Fig 1:**
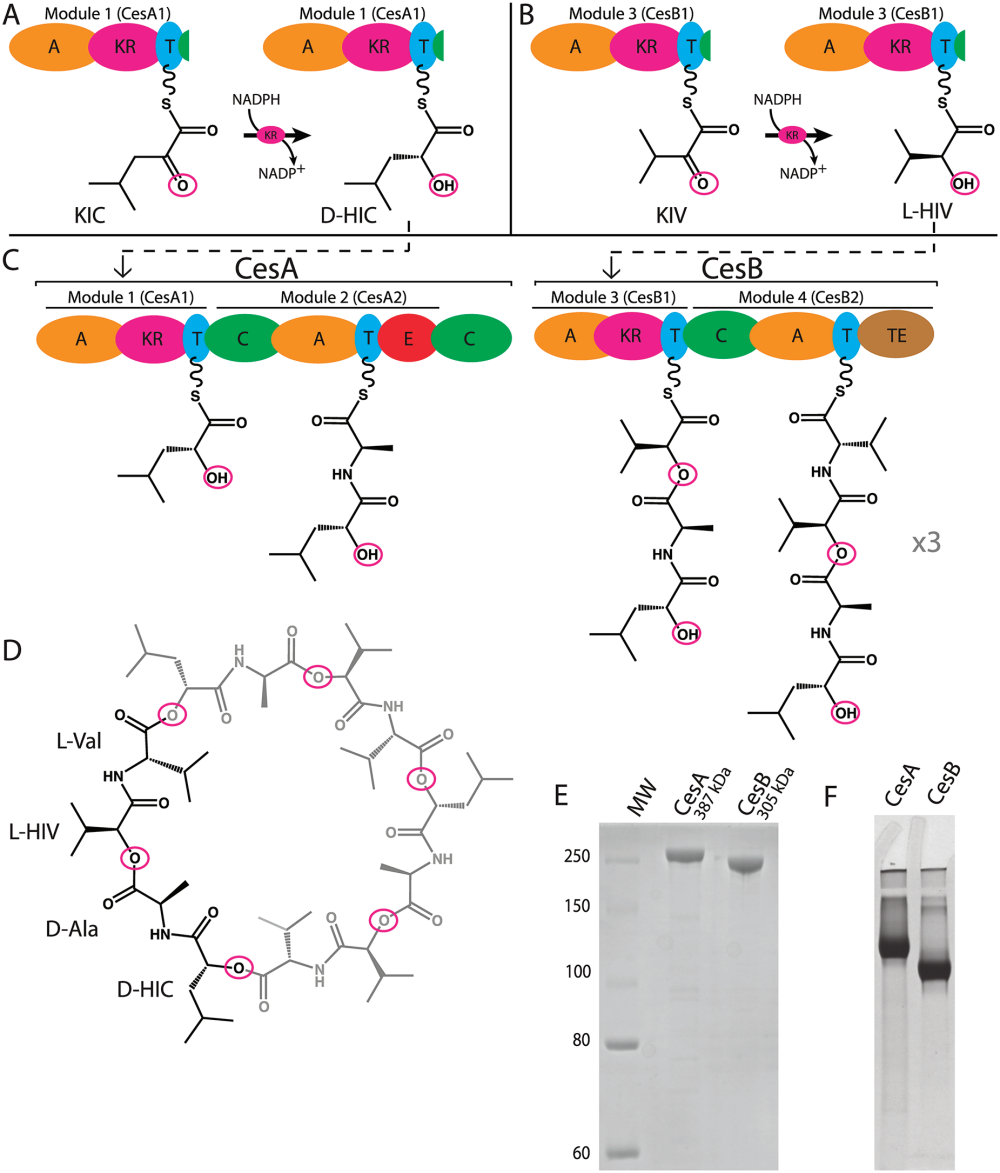
Cereulide synthetase produces the emetic toxin cereulide. (A and B) Modules CesA1 and CesB1 contain KR domains which catalyze the reduction of bound keto acyl groups. (C) Schematic diagram of cereulide synthetase and the synthesis of *D*-HIC—*D*-Ala—*L*-HIV—*L*-Val, which is trimerized to produce mature cereulide (D). (E) Denaturing and (F) native PAGE of NRPS proteins CesA and CesB. CesA and CesB migrate slightly faster than expected from their molecular masses. Domain abbreviations: A, adenylation; KR, ketoreductase; T, thiolation; C, condensation; E, epimerization; TE, thioesterase.

Cyclic depsipeptides are natural products made of diverse acyl moieties linked by amide and ester bonds (Fig 1C and 1D) which have a broad range of biological and medical activities. Cyclic depsipeptides include ionophores, quorum sensing modulators, toxins and antibiotics [1–3]. Some examples are the anticancer agent valinomycin, the biopesticide bassianolide, the piscicide antimycin, the antihelminthic PF1022A, the anti-fungal kutzneride, and cereulide, on which this work is focused [3–8].

The cereulide toxin (Fig 1D) is the causative agent in severe food poisoning associated with emetic strains of *Bacillus cereus*, which can lead to acute liver failure and death [9–11]. The main mechanism of cereulide poisoning is an emetic syndrome mediated by the serotonin 5-HT3 receptor and by stimulation of the vagus afferent nerve [12]. This toxin also exerts cytotoxicity by disrupting the mitochondrial membrane potential through its K^+^ ionophoric activity [13]. Cereulide is highly resistant to heat, extreme pH, and proteases and remains a health hazard even if the cereulide-producing bacteria are killed by thorough cooking of contaminated food [14]. The native role of the toxin for *B*. *cereus* is likely as a siderophore, as its expression increases the fitness of the microorganism in potassium deprived environments [15].

Emetic *B*. *cereus* strains synthesize cereulide through the action of cereulide synthetase (Fig 1C), a heterodimer of the proteins CesA and CesB [16]. These are non-ribosomal peptide synthetase (NRPS) proteins, modular enzymes that employ assembly-line synthetic mechanisms. Each module of an NRPS adds one monomer to the growing peptide chain. The domain arrangement of a canonical NRPS module, such as module CesB2 (Fig 1C), consists of a condensation (C), an adenylation (A), and a thiolation domain (T). The A domain selects and adenylates an amino acid substrate, then attaches it via a thioester bond to the prosthetic phosphopantetheine arm of the T domain. The T domain then transports the bound substrate to the C domain, where it is incorporated into the growing peptide chain by amide bond formation (Fig 1C; reviewed in [17–19]). Because CesB2 is a termination module, it contains an extra domain not found in elongation modules, the thioesterase (TE) domain, which releases the mature nonribosomal peptide by cyclization or hydrolysis. NRPSs frequently display variations of the canonical domain arrangement, including substitutions of canonical domains, and insertion of tailoring domains [20,21], like the epimerization (E) domain found in CesA2.

Modules CesA1 and CesA2 have a domain arrangement and mechanism exclusive to depsipeptide synthetases [22] (Fig 1A and 1B). Magarvey *et al*. showed that the specialized A domain in these modules selects a specific α-keto acid, which is then ligated to the T domain arm [22]. The T domain next transports the tethered α-keto acid to a ketoreductase (KR) domain present in the module. This ~45 kDa domain is embedded in a mobile hinge of the A domain and catalyzes the stereospecific reduction of the α-keto acyl group to an α-hydroxy acyl group, using NADPH as a redox cofactor. The α-hydroxy-acyl-T domain is then brought to the C domain, which catalyzes ester bond formation with the upstream aminoacyl-T domain. This strategy for incorporation of α-hydroxy acids is also found in the synthesis of valinomycin, antimycin and kutzneride [3,5,6], while direct selection of α-hydroxy acids occurs in the fungal synthesis of PF0122A and bassianolide [4,23].

Based on the structure of cereulide, the demonstrated substrate of the A domains and the standard NRPS synthetic logic, module CesA1 is assumed to add *D*-α-hydroxyisocaproic acid (HIC), module CesA2 to add *D*-alanine (Ala), module CesB1 to add *L*- α-hydroxyisovaleric acid (HIV), and module CesB2 to add *L*-valine (Val) to form a tetrapeptide intermediate of cereulide (*D*-HIC—*D*-Ala—*L*-HIV—*L*-Val). This tetrapeptide is presumably then passed to a serine residue in the TE domain. A second tetrapeptide is made by the upstream domains and coupled to the first, making an octapeptide, after which a third is made and coupled in the same way. The TE then cyclizes the dodecapeptide intermediate to release mature cereulide, in the manner similar to that described for the biosynthesis of cyclic peptides such as gramicidin S [24] and surfactin [25].

To increase our understanding of the biosynthesis of cereulide, we have expressed and purified cereulide synthetase and performed a biochemical characterization. We have compared the kinetics and side chain specificity of α-keto acid selection to those of amino acid selection, investigated the requirement for an MbtH-like protein for the activity of the NRPS, assayed the effectiveness of vinylsulfonamide inhibitors on ester-adding modules, and performed *in vitro* peptide synthesis assays. This work provides insight into the functioning of all depsipeptide-synthesizing NRPSs, including valinomycin synthetase, kutzneride synthetase and the depsipeptide synthetases of the antimycin family [26,27].

## Results and Discussion

### The cereulide synthetase subunits can be expressed in *Escherichia coli* and purified to homogeneity

We developed robust expression and purification protocols of the intact NRPSs CesA and CesB, and of the excised first modules of CesA and CesB (designated CesA1 and CesB1, see Fig 1A and 1B), which contain the domain sequence adenylation-ketoreductase-thiolation (A-KR-T) [22]. We ensured the physical integrity of the proteins by denaturing and native gel electrophoresis (Fig 1E and 1F), as well as by dynamic light scattering and size exclusion chromatography.

### The apparent Michaelis constants for cognate keto acids and cognate amino acids

**Fig 2:**
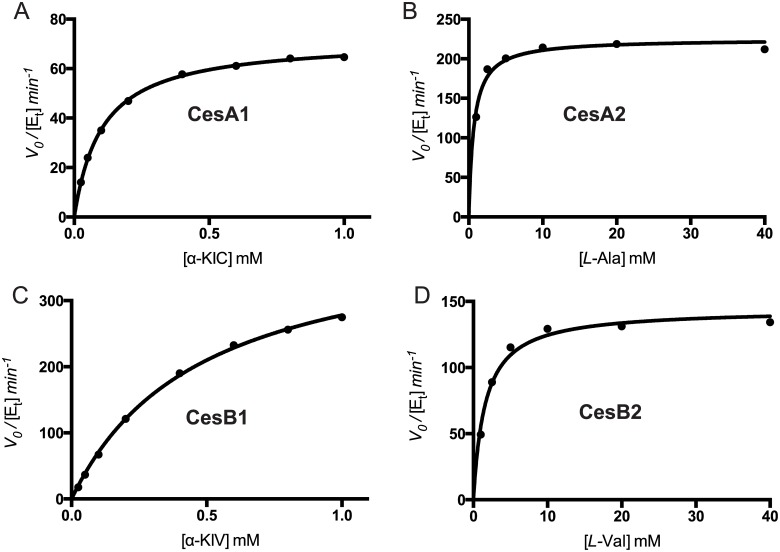
Kinetic characterization of adenylation by A domains using the ATP-PPi exchange assay. Initial velocity versus substrate concentration plots for adenylation of cognate substrates for CesA1 (A), CesA2 (B), CesB1 (C) and CesB2 (D). Curves were fit to the Michaelis-Menten equation. The kinetic parameters obtained are listed in Table 1.

**Fig 3:**
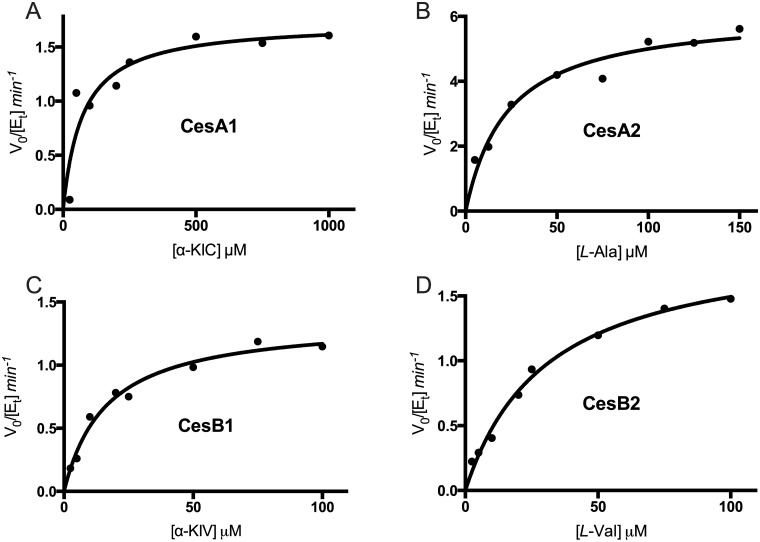
Kinetic characterization of adenylation by A domains using the pyrophosphate production assay. Initial velocity versus substrate concentration plots for adenylation of cognate substrates for CesA1 (A), CesA2 (B), CesB1 (C) and CesB2 (D). Curves were fit to the Michaelis-Menten equation. The kinetic parameters obtained are listed in Table 1.

We performed a kinetic characterization of each of the adenylation domains in CesA and CesB to compare the keto acid—activating A domains to the amino acid—activating A domains. Two commonly used assays for adenylation are a radioactive inorganic pyrophosphate (PPi)—ATP exchange assay [28] and a pyrophosphate production assay [29,30]. The PPi—ATP exchange assay reflects both the forward and reverse rates of the adenylation reaction, as [^32^P]ATP is generated by the reverse reaction using a product (AMP) of the forward reaction and exogenous [^32^P]PPi. In contrast, pyrophosphate production assays reflect only the forward rate because the signal arises from PPi produced during adenylation. It has been reported that two assays give different *k*_*cat*_ and *K*_*m*_ values, but that the apparent *k*_*cat*_ */K*_*m*_ is always similar [29]. We performed both assays with each of the purified proteins and their predicted substrates (Table 1, Fig 2, and Fig 3).

**Table 1:** Apparent catalytic constants of adenylation by cereulide synthetase A domains using the ATP-PPi exchange and pyrophosphate production assays.

Assay	Module	Substrate	*K*_*m*_ (μM)	*k*_*cat*_ (min^-1^)	*k*_*cat*_ */ K*_*m*_ (min^-1^ μM^-1^)
ATP-PPi exchange assay	CesA1	α-KIC	103.0±2.5	71.9±0.4	0.70±0.02
	CesA2	*L*-Ala	673.5±102.6	225.0±5.2	0.33±0.05
	CesB1	α-KIV	479.3±2.5	411.1±9.4	0.86±0.02
	CesB2	*L*-Val	1592±225	144.3±4.3	0.09±0.01
Pyrophosphate-production assay	CesA1	α-KIC	22.4±2.3	3.2±0.1	0.14±0.02
	CesA2	*L*-Ala	23.0±4.9	6.2±0.4	0.27±0.06
	CesB1	α-KIV	16.5±2.6	1.4±0.1	0.08±0.01
	CesB2	*L*-Val	30.9±3.7	2.0±0.1	0.06±0.01

Both assays showed that the enzymes were active in adenylation, with apparent *k*_*cat*_ and *K*_*m*_ values in the range reported for other adenylation enzymes with cognate substrates [29,31,32]. Comparison of the two assays shows no trend with respect to *k*_*cat*_ or *K*_*m*_. Furthermore, while the apparent *k*_*cat*_ */K*_*m*_ of CesA2 with *L*-Ala and CesB2 with *L*-Val are similar across the two assays (CesA2: 0.33 min^-1^μM^-1^ vs 0.27 min^-1^μM^-1^; CesB2: 0.09 min^-1^μM^-1^ vs 0.06 min^-1^μM^-1^), they are not similar for CesA1 with α-ketoisocaproic acid (α-KIC) or CesB1 with α-ketoisovaleric acid (α-KIV) (CesA1: 0.70 min^-1^μM^-1^ vs 0.14 min^-1^μM^-1^; CesB1: 0.86 min^-1^μM^-1^ vs 0.08 min^-1^μM^-1^). There appears to be no fundamental reason that the apparent *k*_*cat*_ */K*_*m*_ should be the same across two disparate assays, which report on different aspects of the reaction (forward reaction vs forward and reverse reaction). Wilson & Aldrich also saw dissimilarity in an A domain [29]. We expect that as more adenylating proteins are evaluated with both methodologies, the previously observed *k*_*cat*_ */K*_*m*_ similarity will be shown to be coincidental.

### An MbtH-like protein is not required for cereulide production

**Fig 4:**
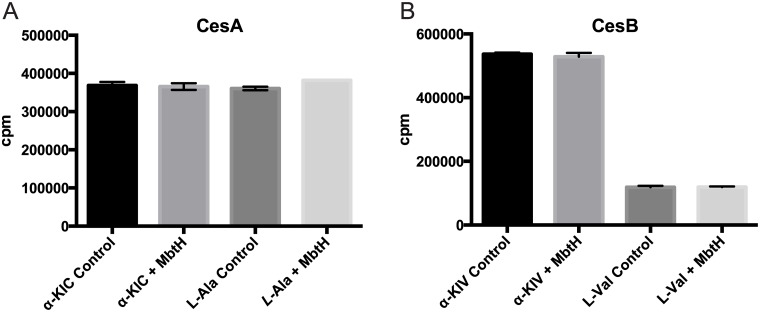
The MbtH-like protein of *B*. *cereus* does not affect the adenylation reactions of cereulide synthetase. The adenylation reaction of each A domain in CesA (A) and CesB (B) with the cognate substrate was evaluated by the ATP-PPi exchange assay at a single point of substrate, with or without MbtH in the reaction, in triplicate. Data is expressed in counts per minute (cpm).

We evaluated whether an MbtH-like protein is required for full adenylation activity in cereulide synthetase, as is the case with some other NRPSs [33,34]. No MbtH-like protein is encoded in the *ces* operon [16], but *B*. *cereus* always contains an MbtH-like protein in the genome. We assayed the activity of each adenylation domain in CesA and CesB in the presence and absence of purified *B*. *cereus* MbtH-like protein. No enhancement of the adenylation activity was observed for any A domain in CesA or CesB (Fig 4), and we conclude that an MbtH-like protein is not involved in cereulide biosynthesis.

### CesA1 and CesB1 display keto acid side chain selectivity

**Fig 5:**
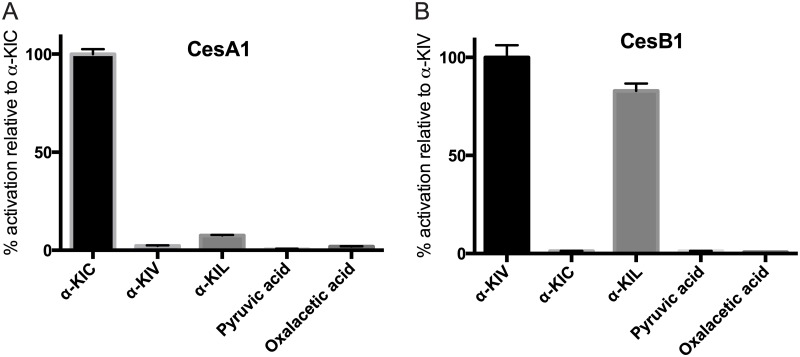
Evaluation of α-keto acid side chain selectivity of cereulide synthetase. The ATP-PPi exchange assay was performed with CesA1 (A) and CesB1 (B) and various α-keto acids as substrates, in triplicate. See S1 Fig for a comprehensive list of the monomers, their abbreviations, and their structures. Data was normalized to the activity obtained with the cognate substrate.

The specialized A domains in CesA1 (Fig 1A) and CesB1 (Fig 1B) have selectivity towards an α-keto over an α-amino or an α-hydroxy group in their substrates [22]. However, the side chain selectivity of the α-keto acid-selecting modules had not yet been probed. We assayed the adenylation activity of CesA1 and CesB1 with α-keto acids that contain side chains present in common amino acids (Fig 5, and S1 Fig): α-KIC, α-KIV, α-ketoisoleucine (α-KIL), pyruvic acid and oxaloacetic acid, which are the keto acid versions of leucine, valine, isoleucine, alanine and aspartic acid respectively. CesA1 shows high side chain selectivity (Fig 5A), whereas CesB1 can activate its canonical substrate α-KIV and can also activate α-KIL (Fig 5B).

That CesB1 can adenylate α-KIL was not unexpected. Pitchayawasin *et al*. reported that *B*. *cereus* produces homologues of cereulide which contain an α-HIC at the position usually occupied by α-HIV (Fig 1D) [35], but the same data would be explained by the presence of α-hydroxyisoleucine (α-HIL), an isomer of α-HIC. In our assay, CesB1 selects α-KIL, but not α-KIC; we propose that this cereulide homologue contains an α-HIL derived from an α-KIL substrate (Fig 5B). Whether the lower selectivity of CesB1 is advantageous for producing cereulide homologues with altered activity is of ongoing interest. Some synthetically produced homologues of cereulide show a marked decrease in toxicity towards HEp2-cells while keeping their ionophoric activity [36], illustrating the potential utility of homologues of cereulide.

### Depsipeptide synthetases can be inhibited by vinylsulfonamide inhibitors

The KR domain is inserted into a loop near the C-terminus of the A domain [22], but it is unclear how it is spatially accommodated into the module or what domain rearrangements must take place during α-keto acid processing. Structural studies could address these questions, but obtaining X-ray structures of multidomain NRPS constructs is challenging, due to their flexibility and intrinsic conformational heterogeneity. Successful structural studies have typically relied on preparing conformationally homogeneous protein by either mutating the conserved serine of the T domain [37] or by locking the enzyme in a single conformation with mechanism based inhibitors [38–40]. Aminoacyl-vinylsulfonamide adenylate analogues have enabled structure determination of A-T didomain constructs, providing valuable information about conformational changes in the adenylation-thiolation cycle [38,39].

**Fig 6:**
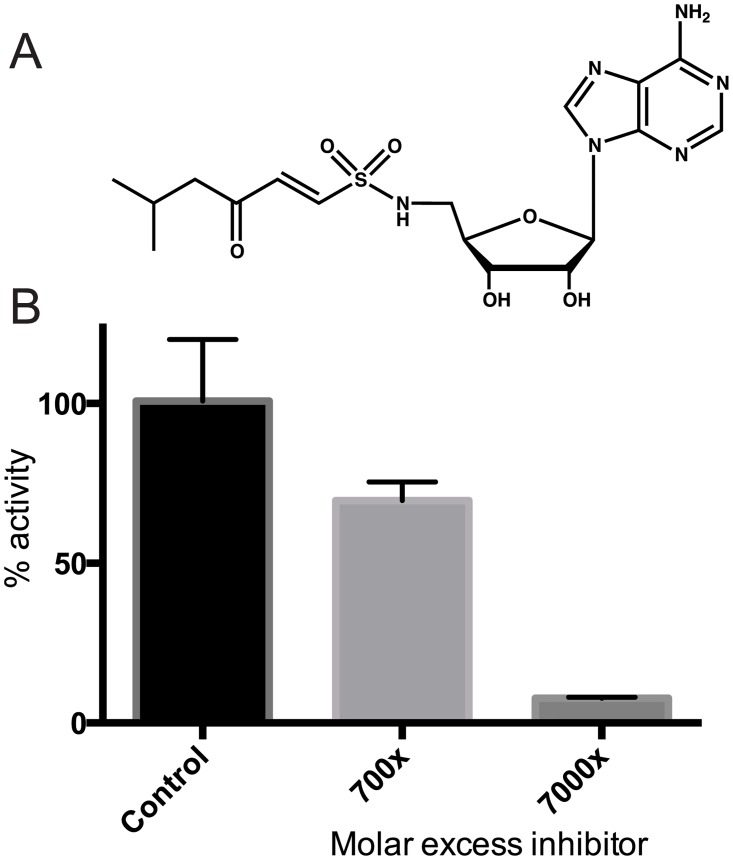
Vinylsulfonamide compounds inhibit α-keto acid adenylation. (A) Structure of the vinylsulfonamide inhibitor for CesA1, α-hydroxyisocaproic acyl-vinylsulfonamide adenylate. The A domain should catalyze the nucleophilic attack of the pantetheine arm thiol on the vinylsulfonamide analogue, but the adenine analogue should not be released in the reaction, trapping the T domain with the A domain. (B) CesA1 is inhibited by the vinylsulfonamide inhibitor. CesA1 was incubated with DMSO or the inhibitor in DMSO. Excess inhibitor and/or DMSO was removed with a desalting column prior to ATP-PPi exchange assay using α-KIC.

We asked whether ketoacyl-vinylsulfonamide adenylate analogues would inhibit cereulide synthetase. CesA1 was incubated with α-hydroxyisocaproic acyl-vinylsulfonamide adenylate (Fig 6A) and its adenylation activity evaluated. The protein could be inhibited by 90%, but this required a vast molar excess of the inhibitor (Fig 6B). Aminoacyl vinylsulfonamide adenylate compounds have been shown to have equally low inhibition efficiencies [41]. The observed inhibition likely resulted from the established mechanism of covalent binding between the α-ketoacyl-vinylsulfonamide and the T domain’s phosphopantetheine arm: Inhibition was not observed with CesA1 that was missing the phosphopantetheine modification because of mutation of the serine attachment site or lack of co-expression with the pantetheinyl transferase Sfp. Stalling of NRPSs using mechanism-based inhibitors is thus not restricted to canonical NRPSs and holds promise to facilitate crystallization of α-keto acid-selecting modules.

### CesA and CesB bind NADPH with a micromolar affinity

**Fig 7:**
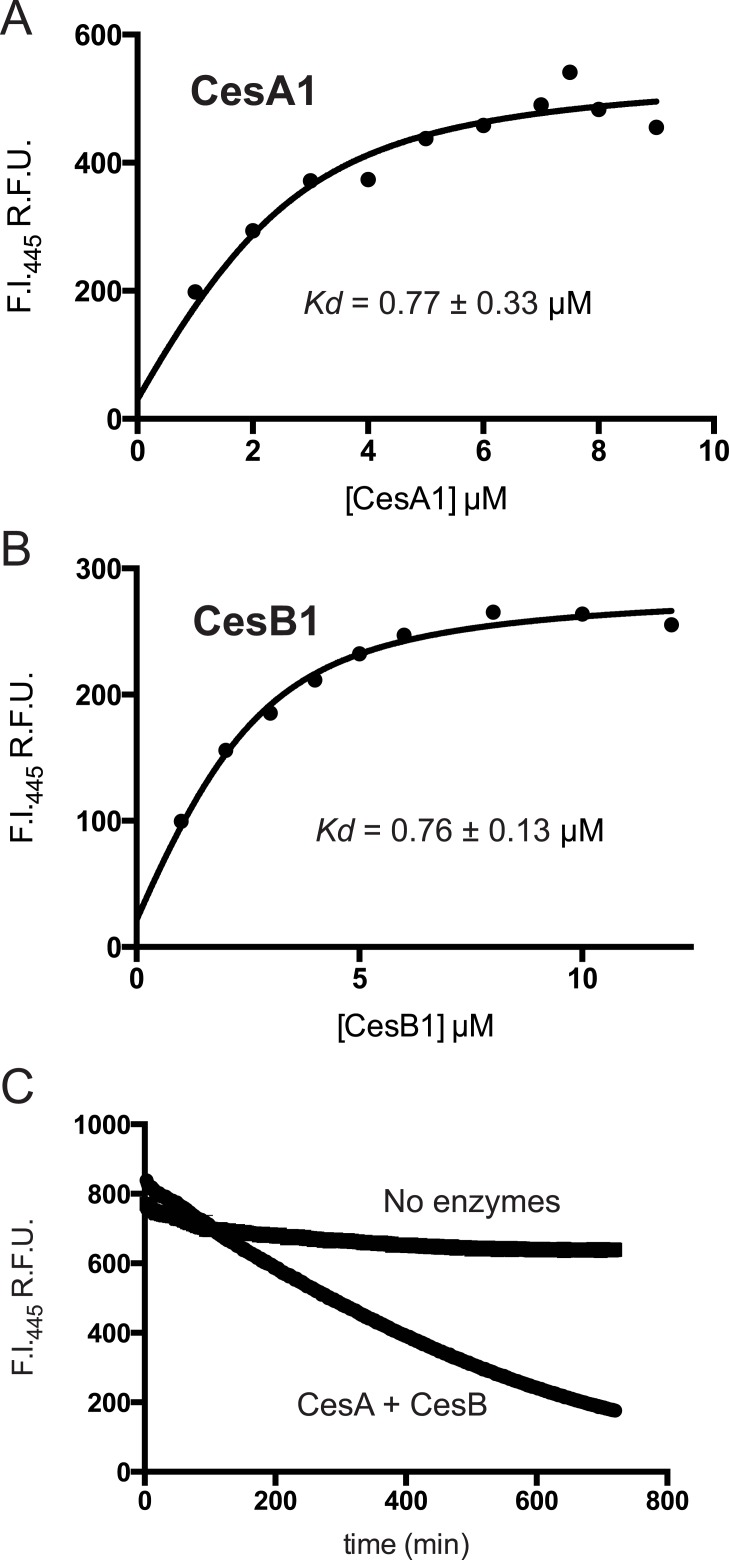
NADPH affinity and turnover in the KR domains in CesA1 and CesB1. (A,B) Fluorescence intensity enhancement is plotted against protein concentration using a fixed concentration of NADPH (2.5 μM); λ_exc_ = 340nm and λ_emm_ = 445nm for CesA1 and CesB1. Curves were fitted to a Morrison equation that includes terms for enhancement of NADPH fluorescence upon binding. (C) Enzymatic NADPH consumption as reported by fluorescence intensity (λ_exc_ = 340nm and λ_emm_ = 445nm).

The KR domains in CesA and CesB catalyze the stereospecific reduction of the α-ketoacyl-T domain to their corresponding α-hydroxyacyl-T domain (Fig 1A and 1B), using NADPH as a cofactor. We assayed NADPH binding to KR domains by fluorescence emission (Fig 7). The *K*_*d*_ was close to 1 μM for both proteins (Fig 7). If the intracellular NADPH concentration in *B*. *cereus* is similar to that in *E*. *coli* (120 μM) [42] or *B*. *subtilis* (44 μM) [43,44], NADPH binding would not be a bottleneck in cereulide synthesis. This *K*_*d*_ value is similar to those found for other ketoreductases, such as the β-ketoacyl reductase domains found in vertebrate fatty acid synthases [45].

### *In vitro* peptide synthesis assays of cereulide synthetase

**Fig 8:**
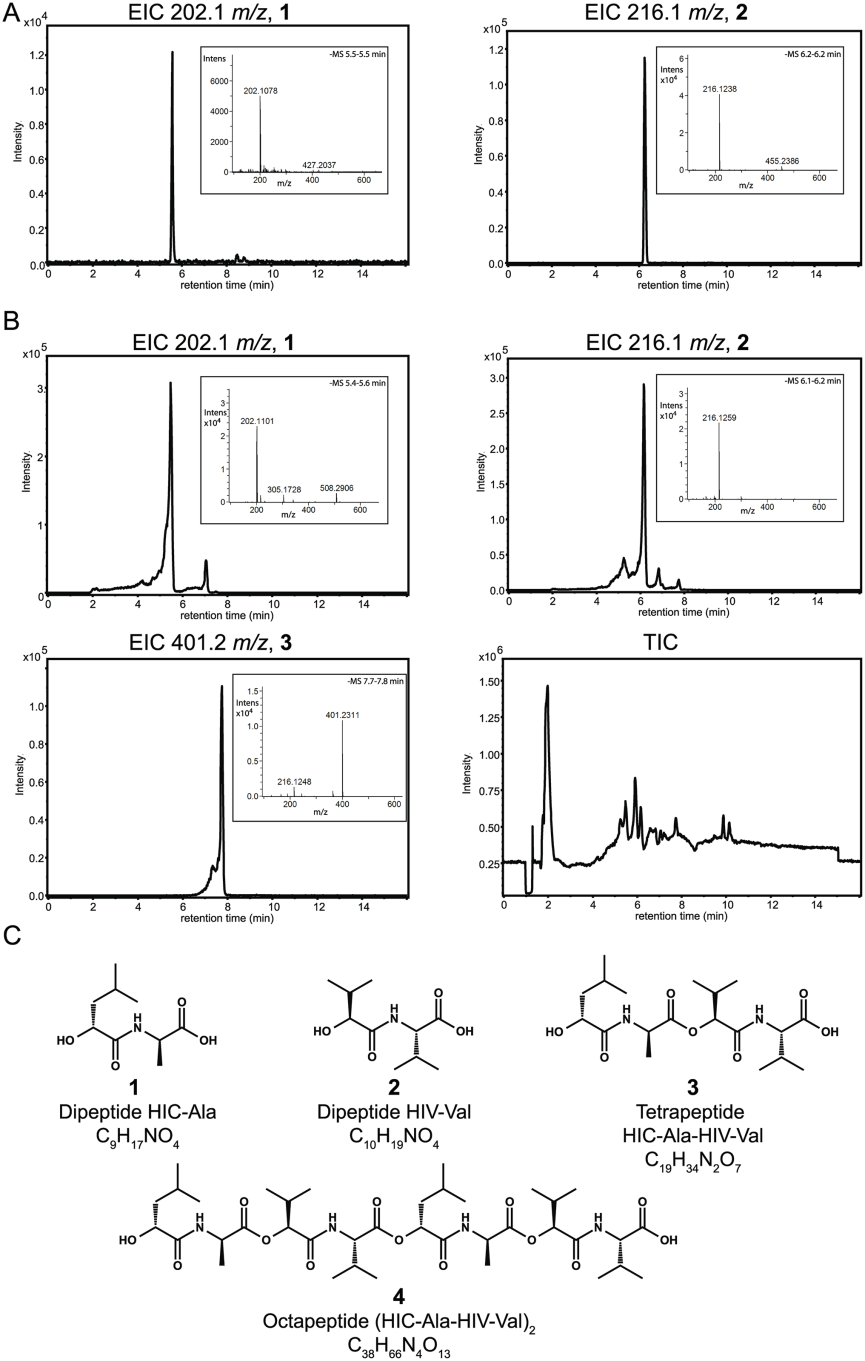
LC-MS analysis of the peptide synthesis reaction of *in vitro-*reconstituted cereulide synthetase. Extracted ion chromatograms (EIC), from (A) synthetic standards of dipeptides **1** and **2**, and (B) from the products of a peptide synthesis reaction of CesA, CesB and substrates. EICs are extracted using exact *m/z* values (±0.05 mass units) calculated from the [M-H]^-^ ions of the compounds shown in (C). Mass spectra corresponding to predominant peaks are shown as insets. (C) Putative chemical structures of the cereulide intermediates detected in the peptide synthesis reaction. The structures are consistent with exact masses and determined molecular formulas, as well as the elution profile of the authentic standards of dipeptides. The shown stereochemistry of the tetra- and octapeptides (**3** and **4**) are consistent with their precursor molecules (**1** and **2**) and their successor molecule (cereulide). However, these putative chemical structures are not absolutely proven here.

We next incubated CesA and CesB with substrates and cofactors *in vitro* and assayed NADPH consumption and peptide synthesis by mass spectrometry. NADPH was consumed when both enzymes were present (Fig 7C), and to a lesser extent by CesA alone. Peptide synthesis and control reactions were then analyzed by HPLC-MS. We could not detect *m/z* peaks corresponding to full-length cyclic cereulide. However, we did detect masses consistent with cereulide precursors dipeptide **1** and dipeptide **2**, which were verified by comparison to authentic standards (CanPeptide Inc, Pointe-Claire, QC, Canada), as well as tetrapeptide **3** (Fig 8) and, in some samples, octapeptide **4** (S4 Fig). Thus, CesA and CesB are working in concert, but not efficiently enough to produce full cereulide against competing rates of hydrolysis (nor to produce octapeptide **4** in every *in vitro* reaction assay). The tetrapeptide **3** and octapeptide **4** precursors are likely lost from the TE domain by hydrolysis before they can be extended to a dodecapeptide and cyclized to cereulide. TE domains are known to have fairly rapid hydrolysis rates *in vitro* [46]. Challenges in getting full-length cereulide production is unsurprising, as few NRPS systems that comprise two or more subunits have been reconstituted *in vitro* [32,47–49] because they are highly complex systems. *In vitro* synthesis of cereulide or any similar depsipeptide is not yet reported, though a related depsipeptide, valinomycin, has been heterologously produced in *E*. *coli* [3,7]. Detection of tetrapeptide **3** and octapeptide **4** provides hope that reaction conditions could eventually be optimized to demonstrate *in vitro* cereulide production.

## Conclusions

The components of cereulide synthetase can be expressed in *E*. *coli* and purified to homogeneity. Upon purification, all adenylation domains remain active and select the monomers predicted from the chemical structure of cereulide, with kinetic constants similar to those of other NRPSs. There are no substantial kinetic differences in activation of keto acids as compared to amino acids. CesA1 shows high side chain selectivity, and CesB1 can efficiently adenylate its canonical substrate α-KIV as well as α-KIL. Cereulide synthetase does not require an MbtH-like protein for its reaction cycle. Vinylsulfonamide inhibitors can inhibit α-keto acid-selecting modules, which could aid efforts to obtain crystal structures of these modules. Together, CesA and CesB were shown to bind and consume NADPH and produce their dipeptides, and the tetrapeptide and octapeptide precursors of cereulide. The characterization of cereulide synthetase presented here and future work with cereulide synthetase could also be relevant for other NRPSs that make useful depsipeptide compounds, such as valinomycin synthetase, antimycin synthetase, kutzneride synthetase, and others yet to be identified.

## Methods

### Reagents

All reagents were purchased from BioShop Canada (Burlington, ON, Canada), unless specified. ATP (A7699) and α-ketoisoleucine (198978) were purchased from Sigma-Aldrich (Oakville, ON, Canada); α-ketoisovaleric acid sodium salt (522198105) and oxaloacetic acid (210056805), from MP Biomedicals (Santa Ana, CA, USA); radioactive inorganic pyrophosphate (NEX-019), from Perkin Elmer (Waltham, MA, USA). All restriction enzymes, T7 DNA ligase and Phusion DNA polymerase were purchased from New England Biolabs (Ipswich, MA, USA). All fast protein liquid chromatography media were purchased from GE Healthcare (Mississauga, ON, Canada) unless otherwise specified.

### Cloning of CesA, CesB, CesA1 and CesB1

Genes for full-length CesA and CesB were cloned from *B*. *cereus* F4810/72 genomic DNA into plasmids pColdI-CesA-Tandem and pColdI-CesB-Tandem. These pColdI-Tandem plasmids encode an N-terminal octa-histidine tag and tobacco etch virus (TEV) protease recognition sequence, and a C-terminal TEV site and calmodulin binding peptide sequence (KRRWKKNFIAVSAANRFKKISSSGAL). The gene sequence for CesA1 (coding for amino acid residues 1–1323) and for CesB1 (amino acid residues 1–1354) were subcloned from the above plasmids into pColdI-Tandem to make pColdI-Tandem-CesA1 and pColdI-Tandem-CesB1. Mutations inserted during the cloning process were corrected by site-directed mutagenesis.

### Protein over-expression and purification

CesA, CesA1, CesB and CesB1 were co-expressed with the promiscuous phosphopantetheinyl transferase Sfp, encoded in a p15A-based plasmid kindly provided by Dr. Christian Chalut and Dr. Christophe Guilhot [50]. Expression of CesA and CesA1 was in *E*. *coli* BL21 (DE3), and of CesB and CesB1, in *E*. *coli* soluBL21 (DE3) (Genlantis, San Diego, CA, USA), grown in LB medium supplemented with 50 μg/ml ampicillin and 34 μg/ml chloramphenicol. For all proteins, cultures were induced at an optical density of ~0.45 with 100 μM isopropyl β-D-1-thiogalactopyranosid (IPTG) and further incubated for 24h or 48h at 16°C. Cells were harvested and resuspended in their buffer A. For CesA and CesA1, buffer A consisted of 50 mM Tris pH 8.0, 250 mM NaCl, 2 mM βME, 2 mM CaCl_2_, 0.1 mM PMSF, 35% v/v glycerol, 10 mM imidazole. For CesB and CesB1, buffer A consisted of 50 mM Tris pH 8.0, 300 mM NaCl, 2 mM βME, 2 mM CaCl_2_, 0.1 mM PMSF, 10% v/v glycerol, 10 mM imidazole. Cells were lysed by sonication, and the lysate was cleared by centrifugation at 18 000 rpm in a JA-25.50 rotor (Beckman-Coulter, Brea, CA). Supernatant was loaded onto a 5ml HiTrap-HP Ni^2+^ column pre-equilibrated with buffer A. Proteins were eluted with buffer which had the same composition but a total of 150 mM imidazole. The eluted protein was then loaded into a calmodulin sepharose 4B column pre-equilibrated with buffer A. The protein was eluted with buffer C, which consisted of 50 mM Tris pH 8.0, 300 mM NaCl, 2 mM βME, 2 mM EGTA, 0.1 mM PMSF and 35% (for CesA1 and CesA) or 10% (for CesB1 or CesB) v/v glycerol. CesB1 and CesA1 elutions were incubated with TEV protease at ratio 1:2 in dialysis against a buffer consisting of 50 mM Tris pH 8.0, 300 mM NaCl, 2 mM βME, 0.1 mM PMSF, 10% v/v glycerol before the sample was reapplied to the calmodulin sepharose 4B column and the Ni^2+^-IMAC column. The flowthrough was loaded onto a HiPrep Sephacryl S-300 HR 26/60 column pre-equilibrated with 25 mM HEPES pH 8.0, 100 mM NaCl and 0.2 mM tris (2-carboxyethyl) phosphine (TCEP). Due to inefficient TEV cleavage, CesA samples used for most of this work were not incubated with TEV protease. Using tagless CesA did not increase the rate of NADPH turnover assays. After the calmodulin column, CesA was dialyzed overnight against 25 mM HEPES pH 8.0, 100 mM NaCl and 0.2 mM TCEP. Enzyme concentration was measured with the Bradford assay. Purified protein was flash frozen in liquid nitrogen and stored at -80°C.

The sequence encoding for the MbtH-like protein from *B*. *cereus* American Type Culture Collection 10987 was synthesized de novo by DNA 2.0 (Menlo Park, CA, USA) and cloned into a pet28-based vector bearing an N-terminal octa-histidine tag and TEV protease recognition sequence. The protein was overexpressed in *E*. *coli* BL21 (DE3) in LB media. Cultures were induced at an optical density of 0.5 with 100 μM IPTG, then further incubated at 16°C for 24h. The protein was purified as described in [51], flash frozen in liquid nitrogen and stored at -80°C.

### ATP-PPi exchange assay

Reactions of volume of 100 μl consisted of 75 mM Tris pH 8.0, 10 mM MgCl_2_, 0.2 mM TCEP, 5 mM ATP, 7% glycerol, 1 mM of unlabelled inorganic pyrophosphate, 0.5 μCi of ^32^P labeled inorganic pyrophosphate, stated concentration of substrate and 20–100 nM enzyme. Reactions were incubated at room temperature for 20 minutes and then stopped with a solution of 1.6% w/v activated charcoal, 4.5% w/v inorganic pyrophosphate and 5% v/v perchloric acid. The resulting suspension was centrifuged at 14 000 rcf, the supernatant was removed, and the pellet was washed twice with 500 μl of 4.5% w/v inorganic pyrophosphate, 5% v/v perchloric acid. The pellet was resuspended in 500 μl of the wash solution and transferred to scintillation vials and 5 ml of scintillation fluid was added. Incorporated radioactivity was quantified in a Perkin Elmer scintillation counter. Total moles of produced ATP were used to calculate *k*_*cat*_ and replotted as a function of substrate concentration. Experiments were performed over multiple substrate concentrations as single experiments and analysed with non-linear regression using the Michaelis-Menten equation in the program Prism 6 (GraphPad Software, San Diego, California).

Triplicate reactions were performed with the MbtH-like protein at saturating concentrations of substrate and a 100x molar excess of purified MbtH-like protein. Absence of YBDZ, the endogenous MbtH-like protein from *E*. *coli*, was confirmed by denaturing polyacrylamide gel electrophoresis analysis and tryptic digest mass spectrometry analysis. Furthermore, CesA1 produced from a ΔYBDZ strain of *E*. *coli* [33], has the same kinetic profile as that from a strain not containing the ΔYBDZ mutation. Keto acid selectivity assays were performed in triplicate with keto acid concentration of 10 mM.

### The pyrophosphate production assay

Pyrophosphate production assay [29] was performed with the EnzChek Pyrophosphate Assay Kit (Invitrogen, Carlsbad, CA). Reactions of 100 μl consisted of 75 mM Tris pH 8.0, 10 mM MgCl_2_, 0.2 mM TCEP, 5 mM ATP, 7% glycerol, 0.2 mM MESG, 1 unit ml^-1^purine nucleoside phosphorylase, 0.03 units ml^-1^ inorganic pyrophosphatase, 150 mM hydroxylamine, varied substrate concentration and 250 nM enzyme. Absorbance at 360 nm was recorded as a function of time in a SpectraMax M5 plate reader (Molecular Devices, Sunnyvale, CA). Slopes were calculated for the linear range of the reaction and converted and plotted as *k*_*cat*,_ as a function of substrate concentration. Experiments were performed over multiple substrate concentrations as single experiments and analysed with non-linear regression using the Michaelis-Menten equation in the program Prism 6 (GraphPad Software, San Diego, California).

### Vinylsulfonamide inhibition assays

α-hydroxyisocaproic acyl-vinylsulfonamide adenylate was synthesized on commission by Zamboni Chemical Solutions, Montreal, QC, Canada. CesA1 (2.8 μM) was incubated with 700× or 7000× molar excess of inhibitor in DMSO, or DMSO control, on ice for 4h, or with 100X molar excess inhibitor or DMSO at 30°C for 6h. After incubation, samples were applied to through a High Trap Desalting column to remove excess unbound inhibitor and/or DMSO. The protein concentration was re-quantified and the PPi-ATP exchange assay was performed in triplicate.

### NADPH binding assays

Reactions of 100 μl volume in 96 well plates (Corning Incorporated, Corning, NY) contained 75 mM Tris pH 8.0, 0.2 mM TCEP, 7% v/v glycerol and 2.5 μM NADPH, and varied concentrations of CesA1 or CesB. After 10 minutes, fluorescence emission spectra were measured using an excitation wavelength of 340 nm and an emission wavelength of 445 nm in a SpectraMax M5 plate reader (Molecular Devices, Sunnyvale, CA). A control without NADPH was performed for each protein concentration. Fluorescence arising from the enzyme controls was subtracted from the observed fluorescence of experimental reactions. The resulting fluorescence at 445 nm was defined as F_mixture_ and replotted as a function of enzyme concentration. Single experiments were performed with multiple enzyme concentration points. The resulting curve was analyzed by nonlinear regression with an equation that encompasses the Morrison equation and the molar ratio of bound NADPH expressed as a function of fluorescence enhancement [45].

### NADPH consumption assay

Triplicate NADPH consumption reactions of 100 μl volume consisted of 75 mM Tris pH 8.0, 0.2 mM TCEP, 10 mM MgCl_2_, 5 mM ATP, 10 mM α-KIC, 20 mM L-Ala, 10 mM α-KIV, 20 mM L-Val, and 100 μM NADPH in black polystyrene 96 well plates for fluorescence detection or 200 μM NADPH in clear polystyrene 96 well plates for absorbance detection. A mixture of CesA and CesB at equimolar concentrations (9 μM) was added and reactions were incubated for 15 minutes to achieve steady state conditions before measuring fluorescence emission at 460 nm using an excitation wavelength of 340 nm, or absorbance at 340 nm, as a function of time. Control reactions lacked keto acids and amino acids, or enzyme. The decrease in absorbance at 340 nm after completion of an independent reaction showed that the observed changes in both fluorescence and absorbance of NADPH are indeed due to enzymatic conversion to NADP^+^.

### Peptide detection by LC-MS

NADPH consumption assay reactions were pooled and methanol was added to a final concentration of 50% v/v. Precipitated protein was removed by centrifugation at 20 000 rpm for 15 minutes. The supernatant was recovered and centrifuged for an additional 15 minutes. LC-MS experiments were performed at the McGill Department of Chemistry Mass Spectrometry Facility (MSF) (Montreal, QC, Canada). Liquid chromatography was performed using a Dionex Ultimate 3000 UHPLC system with a Waters Xterra MS C8 3.5 μm, 2.1×150 mm column at 55°C, a mobile phase A of 200 mM ammonium acetate in water and a mobile phase B of 200 mM ammonium acetate in methanol. Peptides were eluted by a gradient of 90% A to 0% A over 7 minutes at a flow rate of 0.2 ml/min, followed by a further 5 minutes at 0% A. Six minutes of equilibration and one blank injections between samples were performed to ensure that there was no cross contamination. Peptide masses were analysed by an attached Bruker Maxis Impact quadrupole-time of flight (QTOF) mass spectrometer by ESI in negative and positive ionization modes with source nitrogen gas at 250°C and 8 liters per minute. The nebulizer pressure was set at 2.0 Bar psi and capillary voltage was set a 4.5 kV. Data was collected in full scan mode (mass range: m/z 100–2000; scan time: 1 Hz). External calibration was done on each sample from an intra run infusion at the beginning of the each analysis using Agilent ESI tune mix. Data was analyzed using the program Bruker DataAnalysis software. Exact masses were generated with the SmartFormula tool using a 5 ppm mass window. Molecular formulae for each predominant peak were assigned by performing exact mass analysis (Table 2), isotopic pattern analysis, and rbd analysis using the SmartFormula tool (S1 Table). The molecular formulae corresponded to the [M-H]^-^ ions of dipeptide **1**, dipeptide **2**, and tetrapeptide **3** (Fig 8, S1 Table). Furthermore, dipeptides **1** and **2** from the enzymatic reaction elute at the same retention time as their corresponding authentic standards (Fig 8 and Table 2). Authentic standards were synthesized on commission by CanPeptide Inc, Pointe-Claire, QC, Canada, and verified by ^1^H NMR at the Quebec/Eastern Canada High Field NMR Facility (S5 Fig).

**Table 2:** MS analysis of the peptide synthesis reaction of *in vitro-*reconstituted cereulide synthetase.

Sample	Compound	Retention time (min)	Measured m/z	Calculated m/z [M-H]^-^	Error [ppm]
Standards (Fig 8A)	Dipeptide HIC-Ala **(1)**	5.5	202.1078	202.1085	3.5
	Dipeptide HIV-Val **(2)**	6.2	216.1238	216.1241	1.7
Enzymatic reaction (Fig 8B)	Dipeptide HIC-Ala **(1)**	5.4–5.6	202.1101	202.1085	-8.2
	Dipeptide HIV-Val **(2)**	6.1–6.2	216.1259	216.1241	8.2
	Tetradepsipeptide HIC-Ala- HIV-Val **(3)**	7.7–78	401.2311	401.2293	-4.4

Mass spectra were extracted for the predominant peaks in Fig 8. Molecular formulae were assigned by exact mass and isotopic pattern analysis (S1 Table).

## Supporting Information

S1 Fig Abbreviations, names and schematics of the monomers mentioned in the manuscript. (EPS)

S2 Fig Time course of pyrophosphate release for all A domains of cereulide synthetase with their cognate substrates using the nonradioactive pyrophosphate release assay. (EPS)

S3 Fig Additional LC-MS data from analysis of peptide synthesis assay shown in Fig 8. Mass spectra of (A) dipeptide **1** standard, (B) dipeptide **2** standard, (C) dipeptide **1**, (D) dipeptide **2** and (E) tetrapeptide **3**, showing isotopic distribution. (E) Overlay of EICs.
(EPS)

S4 Fig LC-MS of a reaction of *in vitro-*reconstituted cereulide synthetase. (A) Extracted ion chromatograms (EIC) from a reaction of CesA, CesB and substrates. EICs are extracted using the m/z values calculated from the expected molecular masses of peptides. The insets show the mass spectra. (B) Overlay of EICs. (C) Total ion chromatogram (TIC) of the same reaction.
(EPS)

S5 Fig ^1^H-NMR of the authentic dipeptide standards. (PDF)

S1 Supporting information HPLC-MS method for peptide synthesis assay shown in S4 Fig. (DOCX)

S1 Table SmartFormula analysis of mass spectra of putative cereulide precursors. Analysis was performed on the mass spectra shown in Fig 8.
(DOCX)

S2 Table MS analysis of a reaction of in vitro-reconstituted cereulide synthetase. Analysis was performed on the mass spectra shown in S4 Fig.
(DOCX)
