# Supplementary material for: Characterization of Cereulide Synthetase, a Toxin-Producing Macromolecular Machine
Source: PLoS One. 2015 Jun 4;10(6):e0128569. doi: 10.1371/journal.pone.0128569 (PMC4455996; doi:10.1371/journal.pone.0128569)

Water Suppression
10mM dipeptide 1 90% H2O/10% D2O

500 MHz
25C
RT HCN probe
March 6, 2015

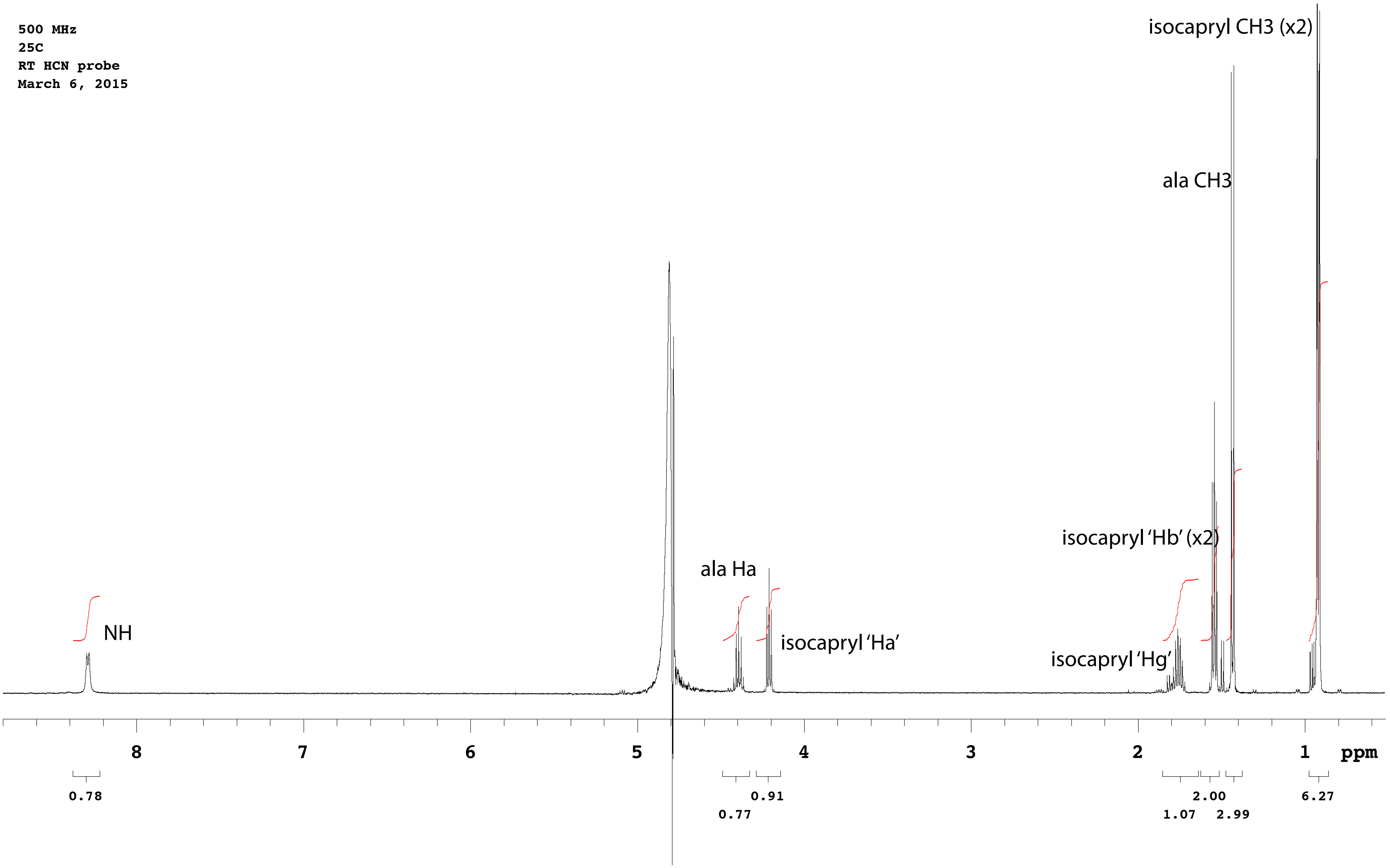

8.3 mM dipeptide 2
90% H2O/10% D2O
25C
500 MHz HCN RT probe
March 11, 2015

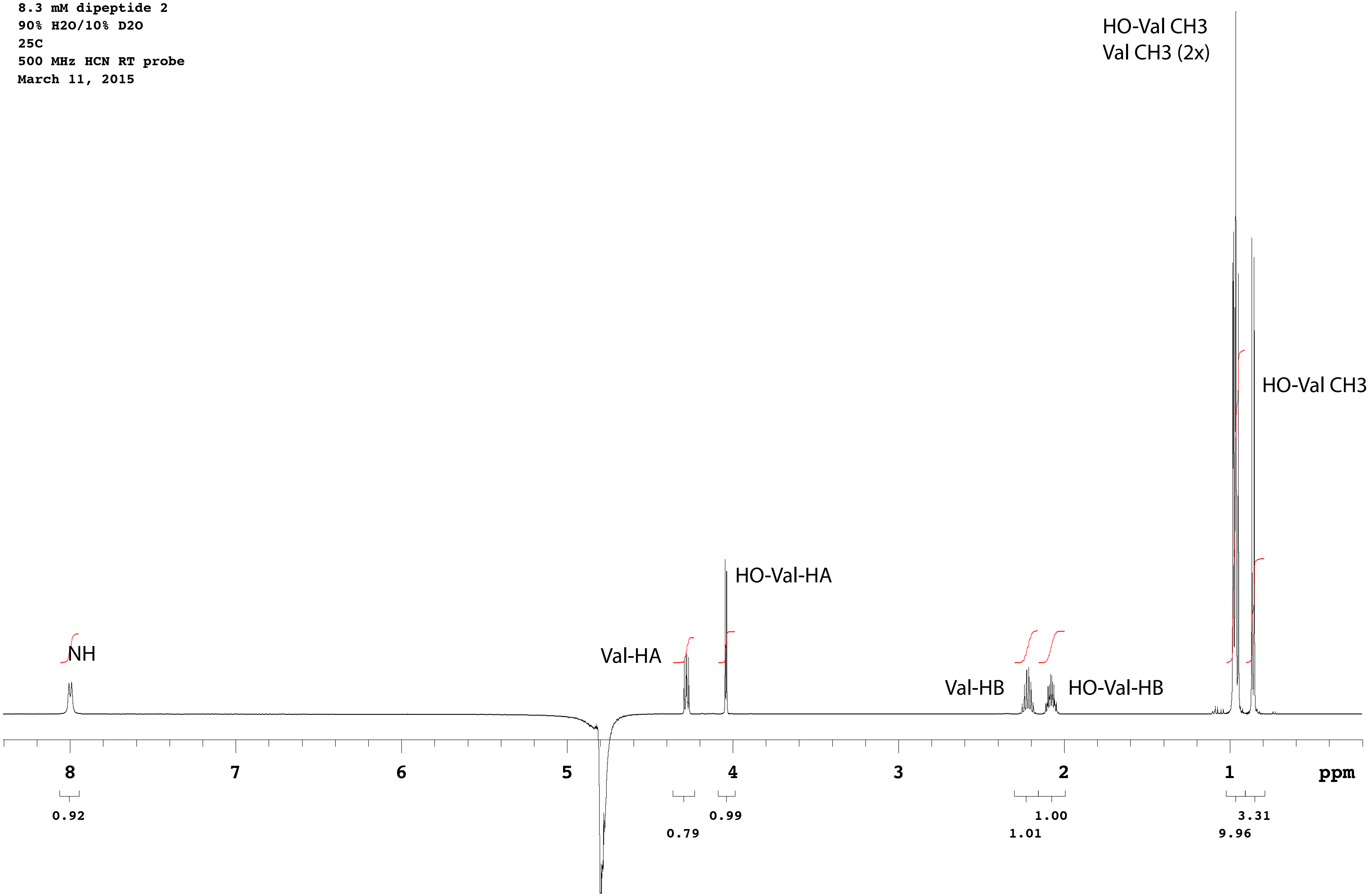

Supplement: S5 Fig — (PDF) [file pone.0128569.s005.pdf]
