# Supplementary material for: Characterization of Cereulide Synthetase, a Toxin-Producing Macromolecular Machine
Source: PLoS One. 2015 Jun 4;10(6):e0128569. doi: 10.1371/journal.pone.0128569 (PMC4455996; doi:10.1371/journal.pone.0128569)
Supplement: S1 Supporting information — (DOCX) [file pone.0128569.s006.docx]

**Supporting Information**

For liquid chromatography and mass spectrometry analysis of the peptide synthesis reaction of *in vitro-*reconstituted cereulide synthetase shown in S4 Fig., which showed masses consent with cereulide precursor dipeptides (1 & 2), tetrapeptide (3) and octapeptide (4), high pressure liquid chromatography and mass spectrometry equipment and media was from Agilent Technologies (Santa Clara, CA, USA), and the experiments were performed at the Rosalind and Morris Goodman Cancer Research Centre Metabolomics Core Facility (Montreal, QC, Canada). Liquid chromatography was performed using a 1290 Infinity ultra-performance UPLC system with a Eclipse Plus C8 1.8 μm, 2.1×100mm column at 55°C, a mobile phase A of 200 mM ammonium acetate in water and a mobile phase B of 200 mM ammonium acetate in methanol. Peptides were eluted by a gradient of 90% A to 0% A over 5 minutes at a flow rate of 0.2 ml/min, followed by a further 5 minutes at 0% A. Six minutes of equilibration and blanks injections between samples were performed to ensure that there was no cross contamination. Peptide masses were analysed by an attached Agilent 6540 UHD Accurate-Mass Q-TOF mass spectrometer equipped with an electrospray ionization (ESI) source in negative ionization mode with source nitrogen gas at 325°C and 9 liters per minute. The nebulizer pressure was set at 45 psi and capillary voltage was set a 4.0 kV. Data were collected in full scan mode (mass range: m/z 50-2000; scan time: 1.4s; data collection: centroid and profile). Data were analyzed using the program Mass Hunter Qual. Calculated exact masses were extracted using a 10ppm window.
