# Supplementary material for: Characterization of Cereulide Synthetase, a Toxin-Producing Macromolecular Machine
Source: PLoS One. 2015 Jun 4;10(6):e0128569. doi: 10.1371/journal.pone.0128569 (PMC4455996; doi:10.1371/journal.pone.0128569)
Supplement: S1 Table — Analysis was performed on the mass spectra shown in Fig 8. (DOCX) [file pone.0128569.s007.docx]

| **Sample** | **Compound** | **Retention time (min)** | **Measured m/z** | **Ion formula [M-H]^-^** | **Calculated m/z [M-H]^-^** | **Error [ppm]** | **Error [mmu]** | **mSigma (#sigma)** | **Score** | **rdb** |
| --- | --- | --- | --- | --- | --- | --- | --- | --- | --- | --- |
| Standards (Fig. 8A) | Dipeptide **1** | 5.5 | 202.1078 | C_9_H_16_NO_4_ | 202.1085 | 3.5 | 0.7 | 11.5 (1) | 100 | 2.5 |
|  | Dipeptide **2** | 6.2 | 216.1238 | C_10_H_18_NO_4_ | 216.1241 | 1.7 | 0.3 | 12.3 (1) | 100 | 2.5 |
| Enzymatic reaction (Fig. 8B) | Dipeptide **1** | 5.4-5.6 | 202.1101 | C_9_H_16_NO_4_ | 202.1085 | -8.2 | -1.6 | 22.1 (1) | 64.19 | 2.5 |
|  | Dipeptide **2** | 6.1-6.2 | 216.1259 | C_10_H_18_NO_4_ | 216.1241 | 8.2 | -1.8 | 21.3 (1) | 62.31 | 2.5 |
|  | Tetrapeptide **3** | 7.7-78 | 401.2311 | C_19_H_33_N_2_O_7_ | 401.2293 | -4.4 | -1.8 | 39.9 (1) | 62.82 | 4.5 |
