# Supplementary material for: Characterization of Cereulide Synthetase, a Toxin-Producing Macromolecular Machine
Source: PLoS One. 2015 Jun 4;10(6):e0128569. doi: 10.1371/journal.pone.0128569 (PMC4455996; doi:10.1371/journal.pone.0128569)
Supplement: S2 Table — Analysis was performed on the mass spectra shown in S4 Fig. (DOCX) [file pone.0128569.s008.docx]

| **Sample** | **Compound** | **Retention time (min)** | **Measured m/z** | **Ion formula [M-H]^-^** | **Calculated m/z [M-H]^-^** | **Error [ppm]** | **Error [mmu]** |
| --- | --- | --- | --- | --- | --- | --- | --- |
| Enzymatic reaction mix (S4 Fig.) | Dipeptide **1** | 4.9 | 202.1092 | C_9_H_16_NO_4_ | 202.1085 | 3.5 | 0.7 |
|  | Dipeptide **2** | 6.2 | 216.1241 | C_10_H_18_NO_4_ | 216.1241 | 0 | 0 |
|  | Tetrapeptide **3** | 5.4-5.6 | 401.2345 | C_19_H_33_N_2_O_7_ | 401.2293 | 12.9 | 5.2 |
|  | Octapeptide **4** | 6.1-6.2 | 785.4608 | C_38_H_65_N_4_O_13_ | 785.4554 | 6.9 | 5.4 |
